# Lower circulating kisspeptin and primary hypogonadism in men with type 2 diabetes

**DOI:** 10.1002/edm2.70

**Published:** 2019-04-21

**Authors:** Henry Asare‐Anane, Emmanuel Kwaku Ofori, Genevieve Kwao‐Zigah, Richmond O. Ateko, Benjamin D. R. T. Annan, Afua B. Adjei, Michael Quansah

**Affiliations:** ^1^ Department of Chemical Pathology, School of Biomedical and Allied Health Sciences University of Ghana Accra Ghana; ^2^ Department of Obstetrics and Gynecology Korle‐Bu Teaching Hospital Accra Ghana

**Keywords:** gonadotrophins, hypogonadism, kisspeptin, testosterone, type 2 diabetes

## Abstract

**Introduction:**

Kisspeptin influence on male androgens is partially understood. We aimed to evaluate serum concentrations of kisspeptin among Ghanaian men with type 2 diabetes and to identify related factors that may contribute to altering circulating kisspeptin.

**Methods:**

A cross‐sectional, observational study. Sixty persons with type 2 diabetes and 60 nondiabetic controls were included in this study. Blood pressure, body mass index (BMI), kisspeptin, luteinizing hormone (LH), follicle‐stimulating hormone (FSH), total testosterone (T), glucose (FBG), glycated haemoglobin (HbA1c) and lipid levels were assessed.

**Results:**

Type 2 diabetic men had lower kisspeptin and T concentrations than controls (*P* = 0.001 for both). Levels of LH and FSH were, respectively, higher in diabetic men compared with their control counterparts (*P* = 0.003; *P* = 0.017). There were negative associations within the diabetic group for kisspeptin vs age (*r* = −0.590, *P* = 0.0001) and kisspeptin vs BMI (*r* = −0.389, *P* = 0.002). Positive associations were also found within the diabetic group for kisspeptin vs T (*r* = 0.531, *P* = 0.001), kisspeptin vs LH (*r* = 0.423, *P* = 0.001) and kisspeptin vs FSH (*r* = 0.366, *P* = 0.004). Lower T (OR = 1.473, *P* = 0.003) and advancing age (OR = 0.890, *P* = 0.004) contributed to decreased kisspeptin levels among Ghanaian males with type 2 diabetes.

**Conclusion:**

Our data demonstrate that circulating kisspeptin and T concentrations are lower among men with type 2 diabetes and highlight the importance of considering kisspeptin concentrations in the management of hypogonadism and type 2 diabetes.

## Graphical abstract

Lower circulating kisspeptin and primary hypogonadism is prevalent in Ghanaian men with type 2 diabetes.

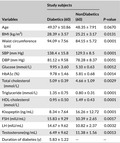

## 1 INTRODUCTION

Kisspeptin hormone, detected in peripheral blood, is found in several organs of the body including the testes, ovary, liver, placenta and the pancreas.^1^ In recent years, kisspeptin effect on reproductive health and metabolism has generated intriguing attention. Biologically, active kisspeptin excites the neurons associated in gonadotrophin‐releasing hormone GnRH) production.^2^, ^3^, ^4^ Loss‐of‐function or genetic changes in the signalling pathway of kisspeptin have resulted in congenital hypogonadotropic hypogonadism CHH) and impaired sexual development.^5^ Kisspeptin receptor knockout mice showed hypogonadotropic hypogonadism HH; however, administration of endogenous GnRH corrected the levels of GnRH suggesting the involvement of kisspeptin in stimulating endogenous GnRH 6, 7 and decisively influencing the pituitary‐gonadal axis.

Increasing changes in human lifestyle over the past years have caused tremendous rise in the number of individuals with type 2 diabetes (T2DM) in Ghana and the rest of the world.^8^, ^9^, ^10^ Uncontrolled T2DM in males are likely to cause serious health complications including loss of libido, erectile dysfunction, impaired sperm production, depressive symptoms, loss of energy, irritability and decreased in cognitive abilities, heart diseases, stroke and chronic inflammation.^2^, ^11^, ^12^

The linkage between kisspeptin and metabolism has not been fully elucidated. Some studies report low endogenous kisspeptin secretion as one of the metabolic and endocrine pathways in the advancement of testosterone deficiency and complications seen in T2DM men.^13^, ^14^, ^15^ However, due to genetic and environmental factors, these results cannot be extrapolated to all populations, especially the African race. A case in point, black African men have higher T concentration than their Caucasian counterpart.^16^, ^17^ This study, thus, aimed to evaluate kisspeptin concentrations in Ghanaian men with T2DM, their relationship with T and identify other clinical and metabolic factors that may influence kisspeptin levels. We hypothesize that men with lower kisspeptin will have lower gonadotrophin and T concentrations.

## 2 MATERIALS AND METHODS

### 2.1 Study design, participants and minimum sample size

This was an observational, cross‐sectional study. Sixty (60) T2DM males, aged between 30‐60 years and attending the National Diabetes Management and Research Centre, Korle‐Bu Teaching Hospital, Accra, were age‐matched with 60 nondiabetic staff/workers of the Korle‐Bu Teaching Hospital, Accra, Ghana. An oral glucose tolerance test (OGTT) was performed on all subjects. All participants gave their consent and answered a validated questionnaire which provided information regarding family history of diabetes, reproductive, socio‐demographic, anthropomorphic and other medical conditions. With a prevalence rate of 8.3% for diabetes mellitus at 95% confidence interval, we established that a minimum sample size of 50 persons was adequate for this study. Patients were excluded if they were on steroid replacement or opiates or on medications that can cause hyperprolactinaemia.

### 2.2 Clinical assessment

Height was measured in centimetres using a wall‐mounted stadiometer (Secca). Body weight (kg) measurement was by a standard digital scale (Tanita Corporation). Body mass index was calculated as weight divided by squared height (kg/m^2^). Blood pressure was taken using a mercury sphygmomanometer and stethoscope after participants had rested for 15 minutes.

### 2.3 Laboratory procedures

Venous blood (8 mL) was collected after an overnight fast (10‐12 hours) for all assays and processed. Serum and plasma were kept at −20°C until required for analysis. Serum kisspeptin, T, LH and FSH were determined by solid phase enzyme‐linked immunosorbent assay (ELISA) (GenWay Biotech Inc). The test utilizes the “sandwich” type enzyme immunoassay that engages a double‐specific monoclonal antibody. A calibration curve was used to determine analyte concentrations from the strength of signal produced. All reactions necessary took place in a coated well and were specific for one type of assay. Lipid profile and FBG were analysed using VITROS system autoanalyzer (Ortho Clinical Diagnostics, version 5, 1 FS). The measurement of ${{\text{HbA}}_{1}}_{c}$ was based on the latex agglutination inhibition assay (Randox Laboratories Ltd).

### 2.4 Statistical procedures

The Statistical Package for the Social Sciences (SPSS) version 20.0 was used for the statistical analysis. Values were expressed as mean plus/minus standard deviations (mean ± SD). After checking normality and variance by continuous fit and Brown‐Forsythe test, the unpaired Student *t* test was used to compare means of parameters between diabetic and nondiabetic male subjects. Spearman's product moment correlation coefficient (rho) analysis was employed to see the association between numeric variables. Multivariate analysis was used to determine independent contribution of several correlates to the variances of kisspeptin levels among diabetic males. A *P*‐value <0.05 was considered significant.

## 3 RESULTS

One hundred and twenty (60 type 2 diabetic and 60 nondiabetic) males, 30‐60 years old, took part in this study. The clinical and biochemical parameters of the study are provided in Table 1. The means for BMI, waist circumference (WC), FBG, HbA1c, total cholesterol, triglycerides (TG), LH and FSH were, respectively, higher *P* < 0.05) in the diabetic group compared with controls. In contrast, kisspeptin and T levels were significantly lower among T2DM men compared with their control counterparts Table 1.

**Table 1:** Clinical and biochemical measurements of study participants

Variables	Study subjects		*P*‐value
	Diabetics (60)	NonDiabetics (60)	
Age	49.37 ± 10.86	48.35 ± 7.91	0.0670
BMI (kg/m^2^)	28.39 ± 3.57	25.21 ± 3.17	0.0131
Waist circumference (cm)	94.09 ± 7.56	84.15 ± 5.72	0.0001
SBP (mm Hg)	138.4 ± 15.8	129.3 ± 8.5	0.0001
DBP (mm Hg)	81.12 ± 9.58	78.28 ± 8.37	0.0051
Glucose (mmol/L)	9.95 ± 3.60	5.10 ± 0.63	0.0012
HbA1c (%)	9.78 ± 1.46	5.81 ± 0.68	0.0014
Total cholesterol (mmol/L)	5.09 ± 0.39	4.66 ± 1.09	0.0029
Triglyceride (mmol/L)	1.35 ± 0.75	0.80 ± 0.31	0.0001
HDL‐cholesterol (mmol/L)	0.95 ± 0.50	1.49 ± 0.43	0.0001
Kisspeptin (ng/mL)	8.34 ± 7.64	16.26 ± 12.72	0.0001
FSH (mlU/mL)	15.83 ± 9.29	10.39 ± 2.65	0.0017
LH (mlU/mL)	14.67 ± 9.62	10.82 ± 2.37	0.0032
Testosterone(ng/mL)	6.49 ± 9.62	11.38 ± 1.56	0.0013
Duration of diabetes (y)	5.83 ± 1.22	—	—

Note

Data presented as mean ± standard deviation (SD).

*P*‐value < 0.05 is statistically significant.

Abbreviation(s): BMI, body mass index; DBP, diastolic blood pressure; FSH, follicle‐stimulating hormone; HbA1c, glycated haemoglobin; HDL, high‐density lipoprotein; LH, luteinizing hormone; SBP, systolic blood pressure.

Association between several correlations with serum kisspeptin, T, LH and FSH were determined and are shown in Table 2. Significant negative associations with kisspeptin were found for age and body mass index (*P* < 0.05) in both study groups. A positive correlation with serum kisspeptin was found for HDL (*r* = 0.362, *P* = 0.005), LH (*r* = 0.432, *P* = 0.001), FSH (*r* = 0.366, *P* = 0.004) and T (*r* = 0.531, *P* = 0.0001) within the diabetic group only. Table 3 shows multivariate analysis of several correlates with kisspeptin. Age (OR = 0.890, *P* = 0.004) and T (OR = 1.473, *P* = 0.003) were independent factors for developing low kisspeptin in T2DM.

**Table 2:** Associations between several correlates and the hypothalamic‐pituitary‐gonadal axis

Variables		Case group (Diabetes (60))				Control group (Nondiabetics (60))			
		Kiss‐P	LH	FSH	T	Kiss‐P	LH	FSH	T
Age	rho *P*‐value	−0.590* 0.001	−0.567* 0.001	−0.354* 0.001	−0.799* 0.001	−0.343* 0.007	−0.397* 0.002	−0.225 0.084	−0.336* 0.009
BMI	rho *P*‐value	−0.389* 0.001	−0.281* 0.030	−0.222 0.088	−0.534* 0.001	−0.600* 0.001	−0.329* 0.010	−0.368* 0.004	−0.134 0.302
SBP	rho *P*‐value	0.008 0.952	−0.226 0.083	−0.074 0.577	−0.051 0.700	−0.074 0.573	0.001 0.991	−0.017 0.896	−0.257* 0.008
DBP	rho *P*‐value	0.092 0.482	−0.208 0.110	−0.117 0.373	−0.069 0.603	−0.120 0.361	−0.120 0.361	−0.164 0.210	−0.184 0.157
Glucose	rho *P*‐value	0.194 0.137	0.225 0.084	0.258* 0.046	0.140 0.285	0.308* 0.017	0.059 0.654	−0.018 0.892	0.009 0.948
HbA1c	rho *P*‐value	0.079 0.549	−0.169 0.197	0.006 0.964	−0.169 0.196	0.383* 0.003	0.131 0.317	0.138 0.294	0.100 0.446
T. Chol	rho *P*‐value	0.115 0.382	−0.108 0.412	−0.099 0.453	0.059 0.656	0.125 0.343	0.343* 0.007	0.464* 0.001	0.087 0.509
TG	rho *P*‐value	−0.162 0.216	−0.228 0.079	−0.177 0.176	−0.236 0.070	−0.127 0.335	−0.046 0.725	−0.057 0.666	−0.080 0.544
HDL	rho *P*‐value	0.362* 0.005	−0.003 0.980	−0.022 0.866	0.229 0.079	0.013 0.921	0.118 0.370	0.230 0.077	0.118 0.370
Kiss‐P	rho *P*‐value	1.000 —	0.432* 0.001	0.366* 0.004	0.531* 0.001	1.000 —	−0.001 0.995	−0.037 0.777	0.075 0.568

Note

*R *is correlation coefficient, *P* < 0.05 is significant. *

Abbreviation(s): BMI, body mass index; DBP, diastolic blood pressure; FSH, follicle‐stimulating hormone; HbA1c, glycated haemoglobin; HDL, high‐density lipoprotein; LH, luteinizing hormone; SBP, systolic blood pressure; T, total testosterone; T. Chol, total cholesterol; TG, triglyceride.

**Table 3:** Risk assessment of serum kisspeptin in study participants

Risk factors	aOR	95% CI	*P*‐value
Testosterone	1.473	1.143‐1.901	0.003*
LH (mlU/mL)	1.055	0.930‐1.159	0.434
FSH (mlU/mL)	1.039	0.930‐1.159	0.500
HDL (mmol/L)	1.886	0.464‐7.662	0.375
Coronary Risk (Ratio)	0.802	0.574‐1.119	0.194
Age (y)	0.890	0.823‐0.963	0.004*
BMI (kg/m^2^)	0.938	0.758‐1.160	0.554

* Statistical significance at *P* < 0.05.

Abbreviation(s): aOR, adjusted odd ratio; CI, confidence interval.

## 4 DISCUSSION

The goal of this study was to evaluate kisspeptin concentrations in Ghanaian subjects with type 2 diabetes and to identify other clinical and metabolic factors that may influence kisspeptin levels. In this study, we found kisspeptin and T concentrations to be lower in T2DM men compared with controls. Related studies targeting middle‐aged men showed that about 25%‐40% of T2DM men have reduced T concentration, resulting in diabetes‐related dysfunction 16, 18 and abnormal functioning of cells in the hypothalamus that secretes luteinizing hormone‐releasing hormone LHRH).^2^, ^19^ This suggests insulin resistance as well as hyperglycaemia could also play a role in the pathogenesis of hypogonadism. This decrease in circulating T, especially in obese diabetic individuals, is related to lower sex hormone‐binding globulin SHBG) levels resulting from decreased hepatic synthesis of this protein.^20^ It is thus hypothesized that steroidal biosynthesis in leydig cells are impaired in the presence of visceral obesity and IR, features seen in T2DM.^16^, ^21^

Additionally, although not completely understood, low T levels, especially those levels seen in obese and overweight men, are reported to be because of increased aromatization to oestradiol (E_2_).^22^, ^23^, ^24^ High circulating levels of E_2_ downregulate the hypothalamic‐pituitary axis and thus the production of T.^16^, ^22^, ^23^

Kisspeptin levels correlated negatively with age of the diabetic subjects. Multivariate analysis further revealed that advancing age and lower T levels independently predicted kisspeptin levels in men. These findings are consistent with prior studies reporting kisspeptin effect in decreasing gonadal function as age advances..^24^, ^25^, ^26^, ^27^, ^28^, ^29^, ^30^

Low levels of kisspeptin have been linked to decreased release of gonadotrophins in males with T2DM.^31^ Interestingly, however, in the case group, LH and FSH concentrations were relatively high, although kisspeptin and T levels were low. It has been observed that low circulating T leads to lack of negative feedback inhibition on LH and FSH secretion, leading to primary hypogonadism.^13^, ^16^, ^32^, ^33^ Further to this, kisspeptin has been suggested to have the ability to increase LH pulsatility and T concentrations in T2DM males with reproductive disorders such as hypogonadism.^14^, ^28^ Dhillo and friends,^27^ initially reported that the administration of exogenous kisspeptin stimulated the hypothalamic‐pituitary‐gonadal axis in human males causing a rise in LH, FSH and T concentrations. Thus, under normal physiology, kisspeptin excites the hypothalamic GnRH neurocytes to produce GnRH into circulation, subsequently stimulating the production of gonadotrophs LH, FSH), which stimulates sex organs in humans to produce T and sperms.^27^, ^34^ Indeed, a novel treatment of kisspeptin administration has been demonstrated to treat infertility in these males with T2DM.^35^ These evidences point to a strong direct influence of kisspeptin in the hypothalamic‐pituitary‐gonadal axis, where adequate levels of circulating kisspeptin are needed to regulate the gonadal‐pituitary axis. Low levels of plasma kisspeptin may serve as a warning sign to the development of low testosterone concentrations in males with T2DM, especially when we consider that these levels have not been established yet in Ghana.

As expected, levels of BMI, waist circumference, FBG and HbA1c were higher in the case group compared with controls. This is supported by a WHO report stating that about 90% of individuals that develop T2DM have excess body weight.^10^ Consistent with the American Diabetes Association, impaired glucose control, as evidenced in T2DM, tends to increase blood concentrations of sugar, which increases the exposure of glucose to certain proteins leading to increased formation of glycated derivatives.^36^ The direct impact of kisspeptin on metabolism has only recently been recognized with kisspeptin implicated in regulating glucose homoeostasis. Efforts to understand this potential role for kisspeptin on impaired glucose metabolism have yielded conflicting results with some noting that kisspeptin stimulates glucose‐stimulated insulin secretion 37, 38 and others reporting the opposite.^39^, ^40^ This study did not find any association of FBG or HbA1c with kisspeptin, contrary to an earlier work.^31^

The case group also presented with higher total cholesterol, triglyceride and cardiovascular risk compared with the control group. Nonavailability of glucose to cells hinders the uptake of lipids and lipoproteins further increasing atherogenic development in insulin‐resistant subjects. The observation of negative associations between BMI and cardiovascular risk with kisspeptin in this study is consistent with earlier works.^41^, ^42^ Wu et al suggested that kisspeptin directly stimulate lipid metabolism in the liver.^43^ Further, kisspeptin is reported to take part in the maturation and metabolism of adipocytes, thus directly influencing lipolysis.^44^

This study had some limitations. The authors were unable to measure the insulin‐resistant state of subjects. Since this was a cross‐sectional study, we are unable to determine fully the causality in relationships observed. Considering the key role kisspeptin plays as a regulator of gonadotrophin‐releasing hormone (GnRH) secretion, there is the need for further research into the synergistic effect of plasma kisspeptin and T levels on diabetic complications and erectile dysfunction. The authors were unable to measure sex hormone‐binding globulin (SHBG), a protein that binds to both T and estrogens and determines their amounts available for action on cells. Importantly, prolactin levels were not performed as part of this study, so we cannot rule out the potential contribution of this hormone on kisspeptin levels. Additionally, a bigger sample size and disease duration for future studies is recommended as these will help to define reference ranges in the population under study.

## 5 CONCLUSION

In summary, kisspeptin levels were lower in Ghanaian males with type 2 diabetes. We further demonstrated that lower testosterone and advancing age were independent risk factors in predicting low kisspeptin concentrations in men. Adequate levels of circulating kisspeptin are needed to regulate the gonadal‐pituitary axis. Data from this work could be useful evidence for the management and care of men with hypogonadism. The implications, however, of low kisspeptin levels in men merit further research.

## CONFLICT OF INTEREST

The authors declare no competing financial interest.

## AUTHOR CONTRIBUTIONS

HA‐A and EKO conceptualized and designed the study. EKO, GK and HA‐A wrote the manuscript. ROA, BDRTA, HA‐A and EKO substantially revised the manuscript. GK, ABA and MQ recruited, performed laboratory work and analysed the data. HA‐A and BDRTA supervised GK.

## ETHICAL APPROVAL

The Ethical and Protocol Review Committee of the College of Health Sciences, University of Ghana approved the study (CHS‐Et/M.1‐P4.2/2016‐2017). Written informed consent was obtained from all participants.

## Footnotes

* Statistical significance at *P* < 0.05.
